# Percutaneous Coronary Sinus-Based Mitral Valve Repair Differentially Modulates Coronary Sinus to Mitral Valve Annulus Geometry and Topography

**DOI:** 10.3389/fcvm.2021.678812

**Published:** 2021-07-15

**Authors:** Dennis Rottländer, Martin Saal, Miriel Gödde, Alev Ögütcü, Hubertus Degen, Michael Haude

**Affiliations:** ^1^Department of Cardiology, Rheinland Klinikum Neuss, Neuss, Germany; ^2^Department of Cardiology, Electrophysiology and Rhythmology, Krankenhaus Porz am Rhein, Cologne, Germany; ^3^Department of Cardiology, Faculty of Health, School of Medicine, University Witten/Herdecke, Witten, Germany

**Keywords:** mitral valve annuloplasty, CT angiography, carillon device, coronary sinus, echocardiography

## Abstract

**Objectives:** Coronary sinus (CS) based mitral annuloplasty using the Carillon device is a therapeutic option for the treatment of functional mitral valve regurgitation (FMR).

**Background:** Little is known about the change of CS and mitral valve annulus (MVA) planes following Carillon implantation and how they are modulated by the tension applied on the device.

**Methods:** In a retrospective single-center analysis, 10 patients underwent Carillon device implantation and received CT-angiography (CTA) prior and post CS based percutaneous mitral valve repair. Patients were assigned to responders or non-responders according to the 3-month transthoracic echocardiographic follow-up. A prototype software was used to assess distance and angulation of both CS (pre) or Carillon-device (post) and mitral annulus planes.

**Results:** Comparison of the distance and angulation of the CS plane or Carillon device plane and the MVA plane prior and post intervention showed significant reduction of distance and unchanged angulation in responders while angulation was increased and distance reduced in non-responders without statistical significance. Furthermore, in FMR responders MVA perimeter, anterior-posterior diameter, intercommisural diameter and MVA area were decreased following successful indirect mitral valve annuloplasty, while in FMR non-responders Carillon device implantation had no effect on MVA geometry.

**Conclusions:** Insufficient reduction of FMR following indirect mitral valve annuloplasty is associated with device malposition in relation to the mitral valve annulus. Patient selection using CTA-derived distance and angulation of CS to MVA planes is one option to increase effectiveness of indirect mitral valve annuloplasty.

## Introduction

Coronary Sinus (CS) to Mitral Valve Annulus (MVA) topography affects reduction in functional mitral regurgitation (FMR) echocardiographic parameters following percutaneous mitral valve repair using the Carillon Mitral Contour System (Cardiac Dimensions, Kirkland, WA, USA) in patients with heart failure (1, 2). Therefore, CT-derived CS to MVA distance and angle seem to be important parameters for patient selection prior Carillon device implantation. A distance <7.8 mm from CS plane to MVA plane and an angulation <14.2° of these planes predicted a reduction of FMR following indirect mitral valve annuloplasty (2). Recently we reported for the first-time results of CT-Angiography (CTA) after Carillon device implantation without a reduction of FMR. Both distance and angulation were in the predictive range of an unfavorable echocardiographic outcome after Carillon implantation (3). Little is known about the change of CS and MVA planes following Carillon implantation and how they are modulated by the tension applied to the device. Different scenarios of baseline topography exist: (i) favorable angle and distance, (ii) favorable angle and unfavorable distance, (iii) unfavorable angle and favorable distance, (iv) unfavorable distance and angle. The influence of these baseline values on the final localization of the Carillon device to date is unknown. Based on the results of CT-based 3D reconstructions of the CS/ great cardiac vein (GCV) and mitral annulus prior and post Carillon device implantation, we aimed to describe the topographical changes of CS and MVA in patients with or without response on FMR in 3 months echocardiographic follow-up after indirect mitral valve annuloplasty. In patients with prior and post CTA, we used a prototype software for CS / GCV reconstruction (3-mensio Structural Heart, Pie Medical Imaging BV, Maastricht, The Netherlands). Beside the topographical relationship of CS and MVA we further aimed to investigate the MVA geometry using intercommisural (CC) diameter, anterior to posterior (AP) diameter, perimeter and mitral valve annulus area as previously reported (4). Therefore, we analyzed for the first time the change of the MVA due to Carillon device implantation using CT-derived D-shaped mitral annulus dimensions in patients with CTA prior and post Carillon device implantation.

## Materials and Methods

### Study Participants

This retrospective study comprises 10 consecutive patients undergoing percutaneous mitral valve repair using a Carillon Mitral Contour System (Cardiac Dimensions, Kirkland, WA, USA) with 3 months transthoracic echocardiography follow up and CTA prior and post intervention. The study was performed in compliance with the Helsinki declaration. An individual written consent was obtained by every patient.

### Carillon Mitral Contour System

Patients with symptomatic (>NYHA II) either heart failure with reduced ejection fraction (HFrEF and HFmrEF, left ventricular ejection fraction <50%) or heart failure with preserved ejection fraction (HFpEF, left ventricular ejection fraction >50%, NT-proBNP ≥125 pg/ml, left atrial dilatation ≥34 ml/m^2^, echocardiographic signs of diastolic dysfunction e.g. E/e' ≥ 13) suffering at least from moderate FMR were eligible for indirect mitral valve annuloplasty (5). Patients with degenerative mitral regurgitation were excluded. In brief CS-based indirect mitral valve annuloplasty was performed using a device based on two self-expanding nitinol anchors connected by a nitinol curvilinear segment. After unsheathing the distal anchor in the distal CS, tension is applied to the system resulting in an anterior movement of the posterior mitral leaflet resulting in a reduction of FMR. Afterwards the proximal anchor is fixed near the CS ostium to maintain tension on the system. Intermittent coronary angiography was performed to rule out coronary artery impingement. Qualitative and quantitative mitral valve assessment using transesophageal echocardiography (TEE) during the procedure was used to monitor the procedural result.

### CTA Analysis

All patients underwent baseline CTA for evaluation of coronary artery disease in a period of 3 months prior intervention. CTA post device implantation was performed within the first year after Carillon device implantation at various time points in each patient. This follow-up CT scan was used in 3 patients to follow-up a postprocedural minor compression of the left circumflex artery (Cx) and in the remaining seven patients to re-evaluate coronary artery disease because of signs of myocardial ischemia (either angina or pathologic treadmill testing). For CTA a 256-slice Brilliance iCT scanner (Phillips Healthcare, Cleveland, Ohio) was used in accordance with the Society of Cardiovascular Computed Tomography (SCCT) guidelines (6). A tube current between 200 and 360 mAs at 120 kV was used, adjusting primarily the mAs based on body weight. CT scans were performed ECG-gated in step & shoot technique in all patients. Collimation of CTA was 256^*^0.6 mm and rotation time 0.27 s. Contrast agent (Imeron 350) was administered with a volume of 100 ml (5.7 mL·s-1) followed immediately by a 50 mL saline chaser. Data were reconstructed at 75% of the RR interval, with a slice thickness of 0.5 mm and a reconstruction interval of 0.3 mm.

The 3mensio Structural Heart (prototype version 10.1) is used to plan catheter-based mitral valve interventions (Pie Medical Imaging BV, Maastricht, the Netherlands). Semi-automatic assessment of the mitral valve using multiplanar reconstructions and volume rendering techniques was used to analyze MVA to CS topography (1, 2). Distance and angle of the MVA plane and CS/GCV plane were automatically calculated as previously described (1, 2). In CTA post Carillon implantation, the device inner curvature was manually tracked analogous to the CS resulting in the Carillon plane. Distance and angle of Carillon device to MVA was automatically calculated as previously described (1, 2).

Mitral valve geometry was assessed by cubic-spline-interpolation of 16 seeding points manually set along the insertion of the posterior mitral valve leaflet and along the anterior peak as previously described (4). The lateral and medial fibrous trigones distance was measured (TT distance) after both trigones were manually tracked. MVA area and perimeter were calculated by projection onto the least-squares plane fitted to the 3D MVA contour (4). Adding the TT distance to the perimeter resulted in the total annular perimeter. The septal-to-lateral distance was defined as the projected distance from the TT line to the posterior peak (intercommissural/CC) and the anterior-to-posterior distance (AP) as the diameter perpendicular to the CC distance and parallel to the TT distance transecting the centroid of the MVA.

### Echocardiography

Transthoracic and transesophageal echocardiography (TTE and TEE) studies were obtained using a Philips iE 33 echocardiography system (Philips, Amsterdam, Netherlands). FMR was diagnosed at baseline using TTE and a two- and three-dimensional TEE approach. Vena contracta (VC), proximal isovelocity surface area (PISA), effective regurgitant orifice area (EROA) and regurgitant volume for quantitative mitral valve assessment were assessed according to current guidelines (7). Grading the severity of FMR was performed using a standard classification (8). Patients with improved quantitative FMR-parameters in 3 months transthoracic echocardiography compared to baseline were allocated to the responder group, whereas patients without beneficial effects on these echocardiographic parameters were classified as non-responders. Responders were defined as patients with improvement in echocardiographic parameters (VC, PISA, EROA and regurgitant volume) as well as overall FMR-classification (8). However, peri-interventional color jet area obtained by TEE was used to estimate acute procedural success. We defined non-responders, when at least 3 parameters and FMR-classification remained unchanged after the Carillon device implantation.

### Statistical Analysis

Statistical analysis was performed using PASW statistics 18 software (SPSS, Chicago, USA). All variables were tested for normal distribution with the Kolmogorov-Smirnov test. In the case of normal distribution, the results are given as mean ± standard deviation (SD) or standard error of mean (SEM) as indicated, otherwise as median and 95% confidence interval. Differences between groups and subgroups were evaluated by chi-square-test for discrete variables and student-*t* test for continuous variables. For ordinal data, Mann-Whitney-U test was used. A *p*-value < 0.05 was considered as statistically significant.

## Results

A total of 10 patients with an implantation of a Carillon device into the coronary sinus, 3 months follow-up TTE and CTA prior and post index procedure were enrolled in this retrospective study. Patients without reduction of FMR after 3 months were assigned to the non-responder group, whereas all patients with improvement of VC, PISA, EROA and regurgitant volume were allocated to the responder group. Four patients were assigned to be non-responders (40.0%), whereas 60.0% could be verified as responders due to echocardiographic quantitative assessment. Mean age accounts for 79.8 ± 1.9 in responders and 81.5 ± 2.8 in non-responders. In our patient cohort half of the patients were male in the non-responder group (50.0%) and 67.7% in the responder group. Left ventricular ejection fraction (LVEF) determined by TTE was reduced in both groups (responders 47.0 ± 2.8%, non-responders 47.8 ± 4.6). Furthermore, left ventricular end-diastolic diameter (LVEDD) was elevated in responders and non-responders (56.0 ± 2.6 mm vs. 56.5 ± 2.1 mm). The majority of patients in the responder and non-responder group had atrial fibrillation (83.3% vs. 75.0%). The left atrium was markedly dilated in both groups (responders: 57.3 ± 5.0 ml/m^2^ vs. non-responders 60.0 ± 12.1 ml/m^2^). NT-proBNP was elevated in responders and non-responders (responders: 4410.5 ± 907.9 pg/ml and non-responders: 3943.0 ± 1729.6 pg/ml). Responders were associated with at least moderate tricuspid regurgitation in 50.0% of the patients in comparison to 25.0% in non-responders. All baseline demographic variables are shown in Table 1.

**Table 1:** Patients characteristics.

	**Responder**		**Non-Responder**	
	***n***	**% or Mean ± SEM**	***n***	**% or Mean ± SEM**
Age	6	79.8 ± 1.9	4	81.5 ± 2.8
Male	4	67.7	2	50.0
**Patients' history**				
Arterial hypertension	6	100	4	100
Diabetes mellitus	1	16.7	0	0.0
Previous heart surgery	2	33.3	3	21.4
Ischemic cardiomyopathy	2	33.3	2	50.0
Dilated cardiomyopathy	2	33.3	1	25.0
Diastolic Heart Failure	2	33.3	1	25.0
Atrial fibrillation	5	83.3	3	75.0
Tricuspid Regurgitation	3	50.0	1	25.0
**Clinical presentation**				
Dyspnea	6	100	4	100
NYHA class 2	1	16.7	1	25.0
NYHA class 3	3	50.0	2	50.0
NYHA class 4	2	33.3	1	25.0
**Medication**				
Betablocker	6	100	4	100.0
ACE-inhibitor/AT1-antagonists/Entresto	6	100	4	100.0
Spironolactone/Eplerenone	4	66.6	3	50.0
Diuretics	5	83.3	3	75.0
ASA	1	16.7	8	57.1
Cumarine	1	16.7	0	0.0
DOAK	4	66.6	3	75.0
**Transthoracic echocardiography**				
LVEF (%)	6	47.0 ± 2.8	4	47.8 ± 4.6
LVEDD (mm)	6	56.0 ± 2.6	4	56.5 ± 2.1
LA volume (ml/m^2^)	6	57.3 ± 5.0	4	60.0 ± 12.1
sPAP (mmHg)	6	47.2 ± 3.8	4	50.5 ± 5.7
**Laboratory Results**				
NT-proBNP (pg/ml)	6	4410.5 ± 907.9	4	3943.0 ± 1729.6
Creatinine (mg/dl)	6	1.2 ± 0.1	4	1.2 ± 0.2
Hemoglobin (g/dl)	6	13.3 ± 0.7	4	13.4 ± 0.4

*LVEDD, left ventricular enddiastolic diameter; NYHA, New York Heart Association; DOAK, direct oral anticoagulation; LVEF, left ventricular ejection fraction; LA, left atrium; sPAP, systolic pulmonary artery pressure; ASA, acetylsalicylic acid*.

**Figure 1:**
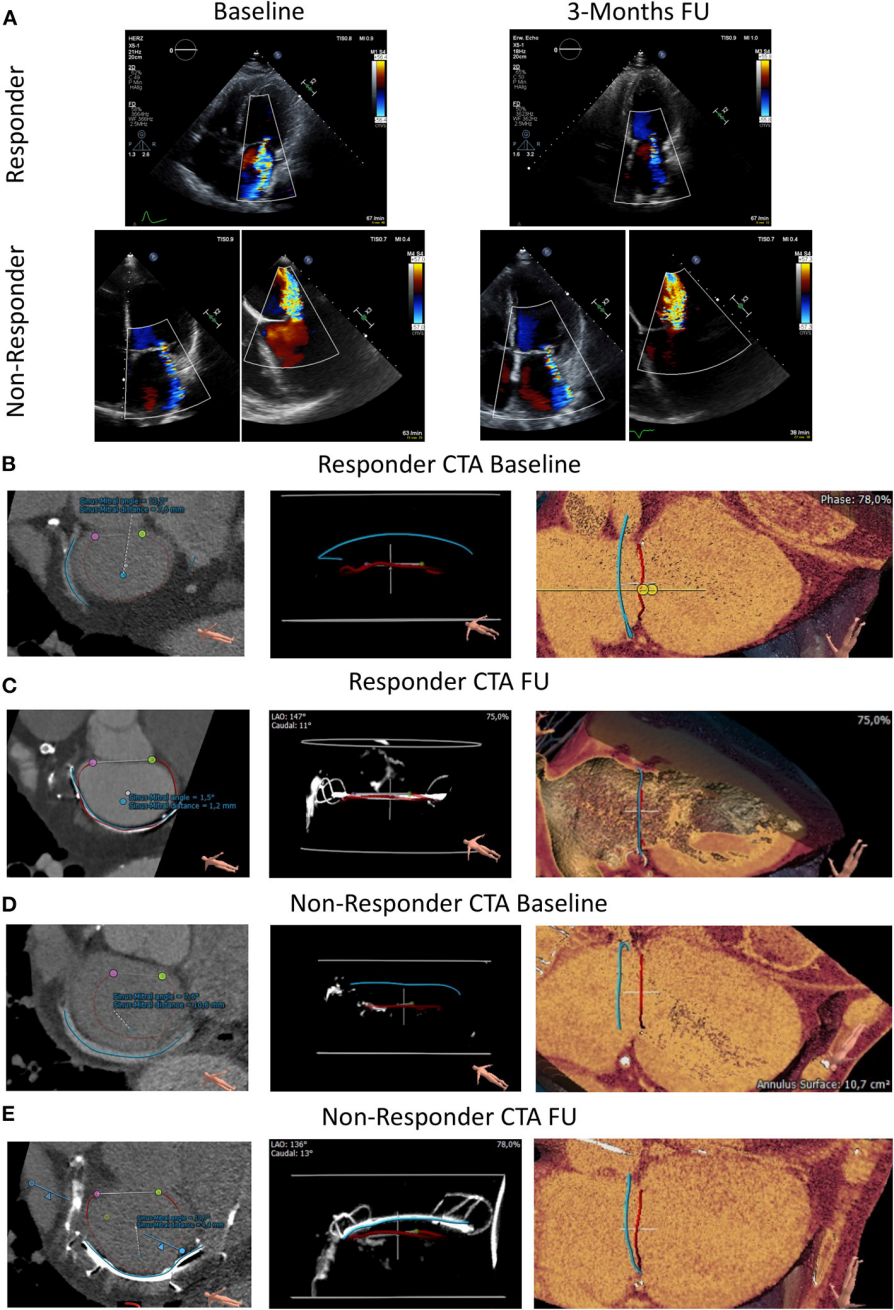
Transthoracic echocardiography and CTA at baseline and 3 months follow-up in an FMR responder and non-responder following Carillon device implantation. **(A)** Transthoracic echocardiographic at baseline and 3 months FU (follow-up) in an FMR responder following indirect mitral valve annuloplasty. Transthoracic and transesophageal echocardiography at baseline and 3 Months FU (follow-up) in an FMR non-responder following Carillon device implantation. **(B, D)** CTA prior index procedure at baseline of a responder **(B)** and non-responder **(D)**. Blue Line: Coronary Sinus; Red Line: Mitral Valve Annulus; Purple and Green Dot: lateral and medial trigone. Distance and angulation of CS to MVA plane as indicated. **(C, E)** CTA post device implantation at follow-up of a responder **(C)** and non-responder **(E)**. Blue Line: inner curvature of the Carillon device; Red Line: Mitral Valve Annulus; Purple and Green Dot: lateral and medial trigone. Distance and angulation of the Carillon device to MVA plane reflects the sinus mitral angle and distance (as indicated).

**Figure 2:**
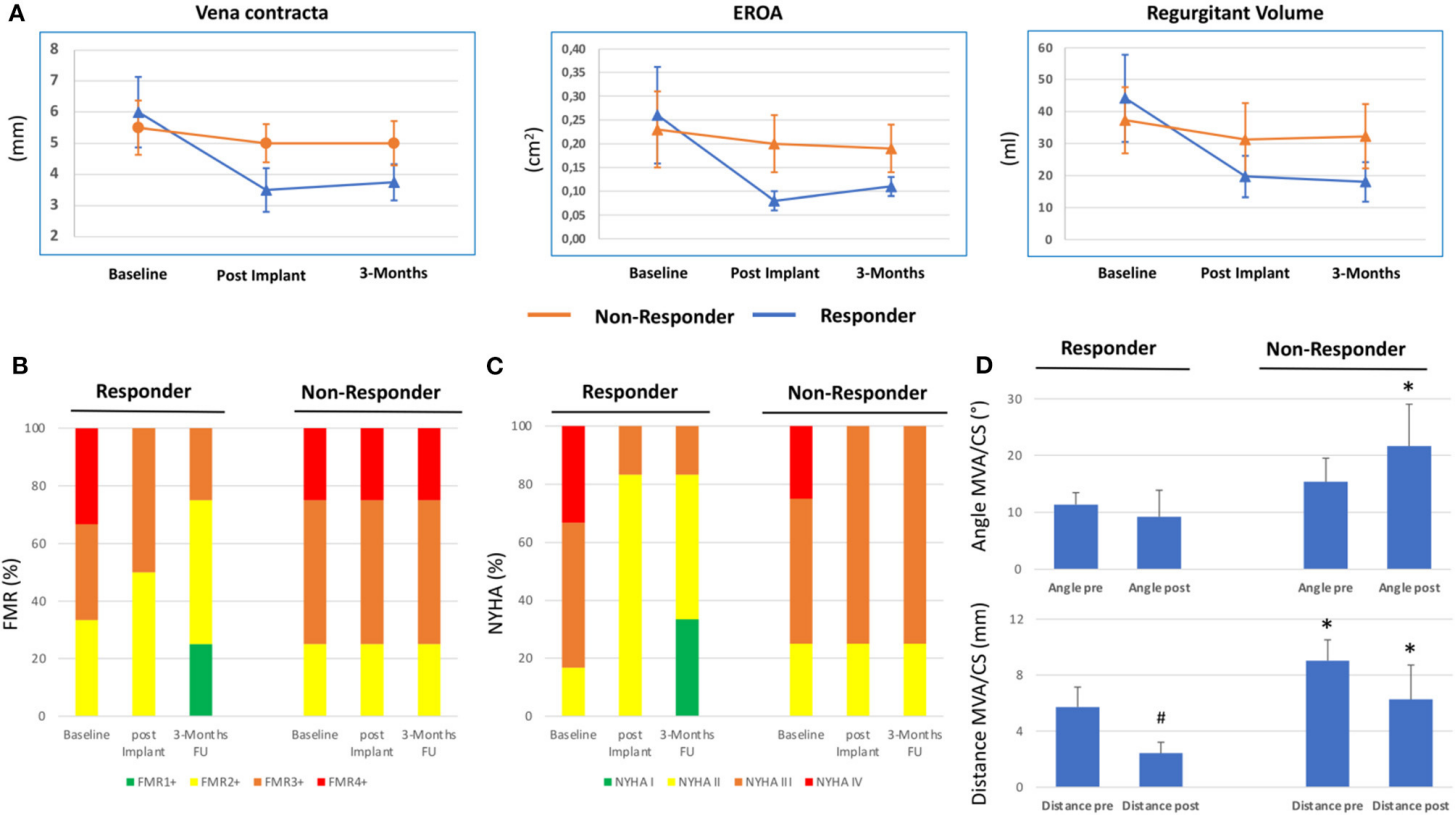
Clinical and echocardiographic response following percutaneous coronary sinus based mitral valve repair. **(A)** Quantitative echocardiographic assessment determining Vena contracta, effective regurgitant orifice area (EROA) and regurgitant volume at baseline, post implantation (implant) and 3 months Follow-up (FU) in responders and non-responders following Carillon device implantation. Mean ± SD. **(B)** Qualitative echocardiographic assessment (relative percentage) at baseline, post implant and 3 months FU in responder and non-responder. **(C)** New York Heart Association (NYHA) classification at baseline, post implant, and 3 months FU in responder and non-responder. **(D)** Distance and angle of coronary sinus (CS)/Carillon device plane and mitral annulus plane (MVA) in responders and non-responders of Carillon device implantation derived from CTA pre and post indirect mitral valve annuloplasty. ^#^*p* < 0.05 vs. pre. *p < 0.05 vs. responder.

Figure 1A shows TTE of FMR at baseline and 3 months follow-up of a responder and non-responder. Over a period of 3 months, quantitative MR assessment showed significant improvement of VC (baseline: 6.0 ± 1.1 mm, 3 months FU: 3.75 ± 0.6 mm), PISA (baseline: 8.83 ± 0.9 mm, 3 months FU: 4.82 ± 0.4 mm), EROA (baseline: 0.26 ± 0.1 cm^2^, 3 months FU: 0.11 ± 0.02 cm^2^) and regurgitant volume (baseline: 44.2 ± 13.7 ml, 3 months FU: 18.0 ± 6.2 ml) in responders but not in non-responders (VC baseline: 5.5 ± 0.9 mm, 3 months FU: 5.0 ± 0.6 mm; PISA baseline: 6.0 ± 1.2 mm, 3 months FU: 6.25 ± 0.8 mm; EROA baseline: 0.23 ± 0.08 cm^2^, 3 months FU: 0.19 ± 0.05 cm^2^ and regurgitant volume baseline: 37.25 ± 10.3 ml, 3 months FU: 32.25 ± 10.0 ml) (Figure 2A). Also, qualitative visual mitral valve assessment and NYHA grade were significantly improved in responders compared to non-responders (Figures 2B,C).

We used a prototype software for CS/GCV reconstruction in CTA to determine the distance and angulation of the mitral annulus and the coronary sinus plane. In CTA post Carillon device implantation, the device was manually tracked and substituted the baseline CS-plane. Figure 1 shows original reconstruction imaging from the 3-mensio structural heart software prior and post intervention, demonstrating the relationship of CS (prior) or Carillon device (post) and mitral valve annulus in a responder and non-responder (Figures 1B,C: responder CTA baseline and follow-up; Figures 1D,E: non-responder CTA baseline and follow-up). Comparison of the distance and angulation of the CS plane or Carillon device plane and the MVA plane prior and post intervention showed significant reduction of distance and unchanged angulation in responders while angulation was increased and distance reduced without statistical significance in non-responders (responder: distance pre: 5.7 ± 1.4 mm and post 2.43 ± 0.8 mm, *p* < 0.01; angle pre: 11.4 ± 2.1° and post 9.2 ± 4.7°, *p* = 0.32; non-responders: distance pre 9.0 ± 1.5 mm and post 6.3 ± 2.3 mm, *p* = 0.1; angle pre: 15.4 ± 4.1° and post 21.6 ± 7.4°, *p* = 0.19; Figure 2D).

**Figure 3:**
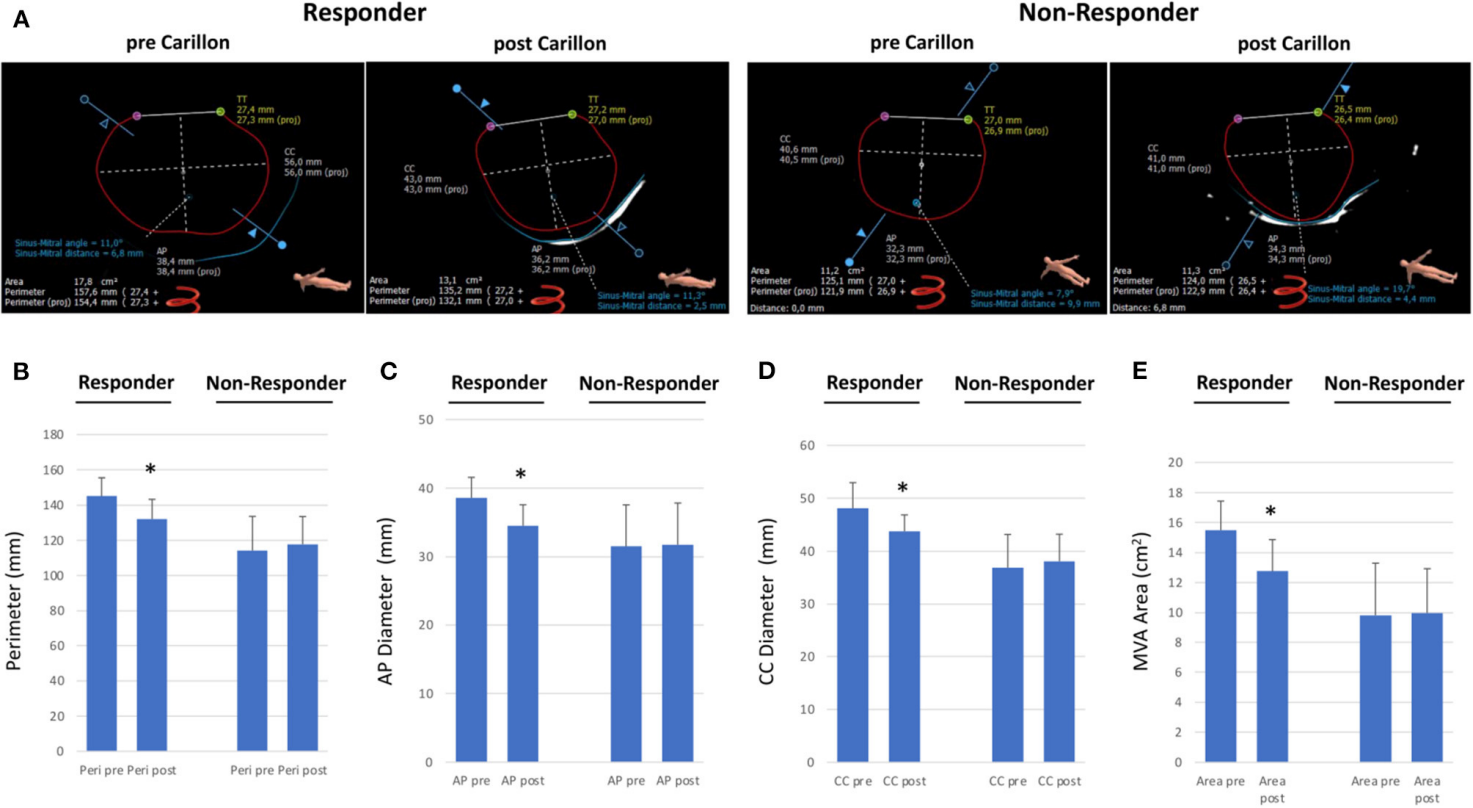
Dimensions of mitral valve annulus in responder and non-responder following Carillon device implantation. **(A)** CTA-derived parameters of mitral valve annulus dimensions (CC = Intercommisural diameter; AP = anterior-to-posterior diameter; TT = trigone-to-trigone diameter) in a responder and non-responder pre and post Carillon device implantation. **(B–E)** Perimeter, Anterior-Posterior diameter, CC diameter and Mitral Valve Annulus (MVA) area in responder or non-responder pre and post indirect mitral valve annuloplasty. *p < 0.05 compared to pre.

To gain insight into the mechanism of modulation of the MVA due to Carillon device implantation, we assessed parameters of mitral annulus geometry prior and post device implantation (Figure 3A). In FMR responders MVA perimeter, AP diameter, CC diameter and MVA area were decreased following successful indirect mitral valve annuloplasty (perimeter: pre 145.1 ± 10.4 vs. post 132.1 ± 11.2, *p* = 0.06; CC diameter: pre 48.1 ± 4.9 vs. post 43.8 ± 3.1, *p* = 0.09; AP diameter: pre 38.6 ± 3.0 vs. post 34.5 ± 3.1, *p* = 0.04; MVA area: pre 15.5 ± 2.0 vs. post 12.8 ± 2.1 mm, *p* = 0.04; Figures 3B–E). In FMR non-responders Carillon device implantation had no effect on MVA geometry (perimeter: pre 114.3 ± 19.3 vs. post 117.7 ± 15.8, *p* = 0.79; CC diameter: pre 36.9 ± 6.3 vs. post 38.1 ± 5.1, *p* = 0.78; AP diameter: pre 31.5 ± 6.1 vs. post 31.8 ± 6.1, *p* = 0.96; MVA area: pre 9.8 ± 3.5 vs. post 10.0 ± 3.0 mm, *p* = 0.95; Figures 3B–E).

**Figure 4:**
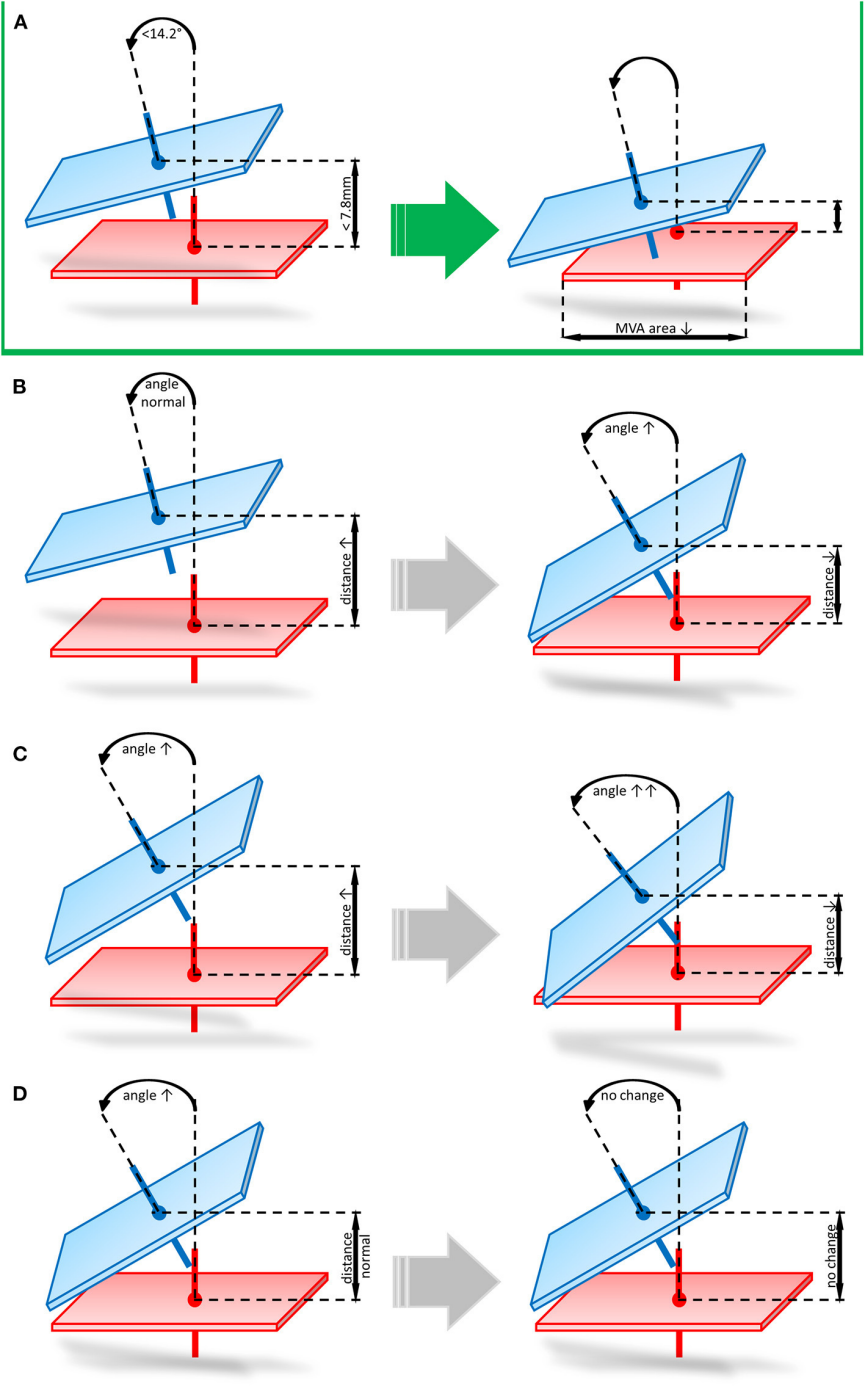
Potential mechanisms of echocardiographic FMR non-response following Carillon device implantation. **(A)** Favorable angle and distance prior to indirect mitral valve annuloplasty leading to reduced distance and unchanged angulation post procedure. **(B)** favorable angle and unfavorable distance (angle < 14.2° and distance > 7.8 mm) leading to decreased distance and markedly increased angulation. **(C)** unfavorable distance and angle (distance > 7.8 mm and angle >14.2°) leading to postinterventional reduced distance and increased angulation. **(D)** unfavorable angle and favorable distance (angle > 14.2° and distance < 7.8 mm) leading to a not significantly changed distance and angle after Carillon device implantation.

In our cohort we found 3 mechanisms of echocardiographic FMR non-response following Carillon device implantation: (i) favorable angle and unfavorable distance (*n* = 2), (ii) unfavorable angle and favorable distance (*n* = 1), (iii) unfavorable distance and angle (*n* = 1). In the first scenario (angle < 14.2° and distance > 7.8 mm) distance was decreased and the angle markedly increased following indirect mitral valve annuloplasty (distance: pre 10.6 mm vs. post 6.4 mm and angle: pre 10.4° vs. 19.7°). When distance is in a favorable range (distance < 7.8 mm) and angle is unfavorable (angle > 14.2°) at baseline, distance and angulation are not significantly changed after Carillon device implantation (distance: pre 7.0 mm vs. post 5.5 mm and angle: pre 20.1° vs. 23.1°). In case both angle and distance are unfavorable (distance > 7.8 mm and angle >14.2°) at baseline, the postinterventional distance is reduced and the angulation further increased (distance: pre 9.3 mm vs. post 6.6 mm and angle: pre 17.1° vs. 30.7°). Figure 4 summarizes these potential mechanisms of non-response.

## Discussion

Indirect mitral valve annuloplasty using the Carillon Mitral Contour System is a therapeutic option to treat symptomatic FMR in heart failure patients (9–11). Successful device implantation improves echocardiographic FMR parameters, heart failure symptoms and quality of life (10–12). Recently, we reported that shorter distance and lower angulation of the CS plane to the MVA plane showed an impact on procedure success up to 3 months follow-up (1, 2). However, the mechanism of interaction between the Carillon device and MVA remained unclear. Furthermore, little is known about the localization of the Carillon device post implantation since tension is applied on the system and fixed by the proximal anchor.

Our small cohort showed comparable baseline echocardiographic results compared to the landmark Carillon trials (9–11). In four patients (40%) Carillon device implantation did not result in an improvement of echocardiographic FMR parameters at 3 months follow-up. Also, in the REDUCE-FMR trial approximately half of the patients did not show reduced FMR severity following indirect mitral valve annuloplasty (9).

In this retrospective analysis, we investigated the impact of Carillon device implantation on CS to MVA topography and MVA geometry using a comparison of baseline and follow-up CTA. Distance and angulation of the CS plane or Carillon device plane and the MVA plane prior and post intervention showed significant reduction of distance and unchanged angulation in responders, while angulation was increased and distance reduced in non-responders without statistical significance. This indicates that the tension applied on the Carillon device per se reduces distance of CS and MVA planes. In case of an unfavorable baseline distance, the tension applied on the system is not sufficient to pull the device toward the MVA plane and reduction of FMR cannot be achieved. Furthermore, in appropriate baseline topography angulation remained mostly unchanged, which is in contrast to non-responders, where the angle tends to increase after Carillon device implantation.

In our cohort we found 3 mechanisms of echocardiographic FMR non-response following Carillon device implantation: (i) favorable angle and unfavorable distance, (ii) unfavorable angle and favorable distance, (iii) unfavorable distance and angle. In the first scenario (angle < 14.2° and distance > 7.8 mm) distance was decreased and the angle markedly increased following indirect mitral valve annuloplasty. This indicates insufficient reduction of device distance to MVA, when baseline distance is unfavorable. Therefore, indirect mitral valve annuloplasty remains unsuccessful and application of tension changes the device angulation without significant reduction of FMR. When distance is in a favorable range (distance < 7.8 mm) and angle is unfavorable (angle > 14.2°) at baseline, distance is slightly reduced and angle slightly increased after Carillon device implantation. This initial state demonstrates, that an unfavorable angle does not allow an application of an effective tension on the MVA resulting in unchanged echocardiographic FMR parameters. In case both angle and distance are unfavorable (distance > 7.8 mm and angle >14.2°) at baseline the postinterventional distance is reduced and the angulation further increased also indicating insufficient tension in case of an unfavorable baseline angle. This data is limited by the small number of patients included in this study, but for the first time we gained insight into the topographical mechanisms of indirect mitral valve annuloplasty using the Carillon device.

Beside CS to MVA topography, we reported D-shaped mitral annulus dimensions for patients prior and post Carillon device implantation. In FMR responders MVA perimeter, AP diameter, CC diameter and MVA area were decreased following successful indirect mitral valve annuloplasty, while in FMR non-responders Carillon device implantation had no effect on MVA geometry. These findings underline the insufficient reduction of MVA in patients with unfavorable baseline CS to MVA topography.

To date several catheter-based techniques to treat FMR exist. One technique is mitral valve edge-to-edge repair. In the COAPT trial FMR reduction was associated with a significantly reduction in hospitalization for heart failure and all-cause mortality compared to medical therapy alone (13). However, for indirect mitral valve annuloplasty no transseptal puncture is needed and overall peri-procedural complication rate is low. Preselection of appropriate CS to MVA topography might further improve echocardiographic and clinical results of indirect mitral valve annuloplasty. Our results in a small cohort should encourage further randomized clinical trials with patient selection using CTA prior Carillon device implantation.

## Conclusions

Insufficient reduction of FMR following indirect mitral valve annuloplasty is associated with device malposition in relation to the MVA. Patient selection using CTA-derived distance and angulation of CS to MVA planes is one option to increase effectiveness of indirect mitral valve annuloplasty. However, our study is hypothesis-generating and needs to be confirmed by larger, randomized, prospective trials.

### Study Limitations

This is a retrospective, non-validated, single-center study with consequent statistical limitations. It enrolls a small number of patients with CT scans pre and post intervention. Therefore, the results should be regarded as hypothesis-generating and needs to be verified by randomized, multi-center, prospective trials with a larger study population.

## Data Availability Statement

The datasets used and/or analyzed during this study are available from the corresponding author on reasonable request.

## Ethics Statement

Ethical review and approval was not required for the study on human participants in accordance with the local legislation and institutional requirements. The patients/participants provided their written informed consent to participate in this study.

## Author Contributions

DR: conceptualization, data collection, analysis, interpretation, drafting, and final approval. MS: data collection, analysis, interpretation, drafting, and final approval. MG and AÖ: data collection. HD: conceptualization and critical revision. MH: conceptualization, data analysis, interpretation, critical revision, and final approval. All authors contributed to the article and approved the submitted version.

## Conflict of Interest

HD is a consultant for Biotronik and Cardiac dimensions. MH received institutional grants/research supports from Abbott, Biotronik and receipt of honoraria or consultation fees from Biotronik, Cardiac Dimensions, OrbusNeich and Philips. The remaining authors declare that the research was conducted in the absence of any commercial or financial relationships that could be construed as a potential conflict of interest.
